# Electrochemical DNA Biosensor Based on Immobilization of a Non-Modified ssDNA Using Phosphoramidate-Bonding Strategy and Pencil Graphite Electrode Modified with AuNPs/CB and Self-Assembled Cysteamine Monolayer

**DOI:** 10.3390/s22239420

**Published:** 2022-12-02

**Authors:** Hamza Moustakim, Hasna Mohammadi, Aziz Amine

**Affiliations:** Chemical Analysis and Biosensors Research Group, Laboratory of Process Engineering and Environment, Faculty of Sciences and Techniques, Hassan II University of Casablanca, P.A. 146, Mohammedia 28806, Morocco

**Keywords:** electrochemical DNA biosensors, pencil graphite electrode, DNA immobilization, phosphoramidate-bonding, methylene blue, differential pulse voltammetry

## Abstract

The present paper describes an alternative approach to the traditionally used covalent immobilization methods that require cost-intensive and complicated chemistry modification of a single-stranded DNA (ssDNA) capture probe. The low-cost pencil graphite electrode (PGE) modified with carbon black (CB) and gold nanoparticles (AuNPs) was used as an electrochemical platform and the non-modified ssDNA was immobilized on a self-assembled cysteamine modified AuNPs/CB–PGE through a phosphoramidate bond between the 5′-terminal phosphate group of ssDNA and the primary amine group of cysteamine. The microRNA-21 was used as a target model in the fabrication of this electrochemical DNA biosensor and the hybridization process with the complementary probe was monitored by differential pulse voltammetry using methylene blue (MB) as an electrochemical hybridization indicator. The decreased reduction peak current of MB shows a good linear correlation with the increased concentration of microRNA-21 target sequences because the MB signal is determined by the amount of exposed guanine bases. The linear range of the fabricated DNA biosensor was from 1.0 × 10^−8^ to 5.0 × 10^−7^ M with a detection limit of 1.0 × 10^−9^ M. These results show that the covalent immobilization of a non-modified ssDNA capture probe through a phosphoramidate-bonding strategy could serve as a cost-effective and versatile approach for the fabrication of DNA biosensors related to a wide range of applications that cover the fields of medical diagnostic and environmental monitoring. The fabricated electrochemical DNA biosensor was used to analyze microRNA-21 in a (spiked) human serum sample and it showed satisfactory and encouraging results as an electrochemical DNA biosensor platform.

## 1. Introduction

The massive quantities of genetic information obtained through modern DNA sequencing technologies and the capability of bioinformatics tools to give us specific base sequences related to some well-known genetic and infectious diseases are very important resources nowadays in medical diagnostic and environmental monitoring [1,2]. Latest progress in transcriptomics technologies and computational strategies allows us to go even farther and integrate a new set of sequences known as non-coding RNA sequences [3,4,5]. These non-coding RNAs play an important role in regulating gene expression at the transcriptional and post-transcriptional levels [6]. The well-characterized non-coding RNAs known as microRNAs are listed in the miRBase database with no less than 4571 entries for the human species and their deregulation is associated with a wide range of diseases such as cancers and cardiovascular diseases [7,8,9,10]. These growing challenges and threats, as well as the established bioinformatics databases, lead to the fast expansion of several detection methods for specific DNA and RNA sequences [11,12,13]. The conventional methods that are commonly used for the detection of specific base sequences are accurate and reliable, but they present some limitations such as technical complexity and the requirement of sophisticated instrumentations [14,15]. These limitations lead to an increasing development of electrochemical DNA biosensors because they provide a simple and low-cost detection method that can be used quickly and efficiently in a point-of-care environment and for a wide range of applications [16,17].

The diversified and innovative approaches for the fabrication of electrochemical sensors and DNA biosensors today offer a promising opportunity to introduce simple and efficient devices for medical diagnostic and environmental monitoring [18,19,20]. The use of pencil graphite electrodes (PGE) gained much attention in recent years as electrochemical sensors and DNA biosensors because of their good electrical conductivity, cost efficiency, large electrode surface area, and ease of availability [21]. Numerous nanomaterials can be used for the modification of PGE because of their unique physical and chemical properties such as platinum, gold, silver, graphene, carbon black, and multi-walled carbon nanotubes [22]. The use of gold nanoparticles (AuNPs) has become of interest because they possess excellent conductivity and high biocompatibility, and they can easily immobilize the thiolated chemical molecules through the Au-S bond [23]. These mentioned advantages are among the reasons leading to an increase in the application of AuNPs in different fields of electrochemical sensors and DNA biosensors [24,25]. On the other hand, carbon black (CB) nanoparticles became widespread because of their good conductivity, as well as their rediscovered cost efficiency (1 €/Kg) [26]. The use of CB is widely shown to be of benefit in several applications compared to other used carbon nanomaterials and its use can be considerable as it can play a significant and impactful role in the development of cheap electrochemical sensors and DNA biosensors [27,28].

The immobilization of a single-stranded DNA (ssDNA) probe on the electrode surface to recognize its complementary target sequence through a hybridization process is the crucial step in the fabrication of electrochemical DNA biosensors [29]. There are several approaches for immobilizing an ssDNA probe such as electrostatic adsorption [30], avidin-biotin interaction [31], and covalent bonding [32]. The covalent bonding of ssDNA to the electrode surface has been widely adopted as the usual method for the preparation of DNA biosensors. This approach is preferred because it allows a high binding strength and good vertical orientation, which can result in high hybridization efficiency with the complementary target sequence [33]. The ssDNA capture probe is usually linked with thiol [-SH] or amine [-NH_2_] groups at the 5′-terminal end to make covalent bonding with metal or specific functional groups introduced to the electrode surface [34,35]. The modification of the ssDNA capture probe with some common functional groups used for covalent immobilization can be, however, a tedious and expensive process. The immobilization of a non-modified ssDNA capture probe as an alternative is usually done by electrostatic adsorption but it has some limitations such as the high risk of desorption from the electrode surface, as well as the random orientation of the immobilized ssDNA capture probe, which can influence the hybridization efficiency with the complementary target sequence [36]. The development of an immobilization strategy based on the covalent bonding of a non-modified ssDNA capture probe can therefore be an interesting and effective alternative to the usual immobilization methods.

Different approaches have been explored for the detection of hybridization between the immobilized ssDNA capture probe and the complementary target sequence. These approaches include direct detection methods based on guanine bases oxidation [37], as well as indirect methods based on nanoparticles [38], enzyme-labels [39], and redox-active indicators [40]. The incorporation of redox-active indicators to the DNA surface is the most popular way to monitor hybridization between the immobilized ssDNA capture probe and the complementary target sequence [41]. These redox-active indicators include metal complexes such as Co(phen)_3_^3+^ and Ru(NH_3_)_6_^3+^ [42,43], anticancer drugs such as daunomycin and doxorubicin [44,45], and some organic dyes such as meldola blue and methylene blue [46,47]. The use of methylene blue (MB) as an electrochemical redox hybridization indicator has become a popular choice because of its great benefits including low required potential and high discrimination of binding affinities between single and double-stranded DNA sequences. The pioneering work on the use of MB as an electrochemical hybridization indicator has been successfully demonstrated by Erdem et al. [48]. Their work reported that the obtained MB current response was higher at the ssDNA-modified electrode in comparison with the dsDNA-modified electrode. The research group concluded that this finding is essentially due to the high affinities of MB to the free guanine bases on ssDNA and that less accessible guanine bases after hybridization because of the formation of DNA duplex leads to a low current response. This conclusion was in agreement with other previous studies and shows that this interaction can be used as an effective approach for the electrochemical monitoring of the hybridization process [49,50,51,52].

Here we present an alternative approach for the fabrication of electrochemical DNA biosensors based on the covalent immobilization of a non-modified ssDNA capture probe from its 5′-terminal phosphate group. The low-cost PGE modified with AuNPs/CB was used as an electrochemical platform and the non-modified ssDNA capture probe was immobilized on a self-assembled cysteamine-modified AuNPs/CB–PGE through a phosphoramidate bond between the 5′-terminal phosphate group of ssDNA and the primary amine group of cysteamine. The microRNA-21 was used as a model in the fabrication of this electrochemical DNA biosensor and the hybridization process with the complementary probe was monitored by differential pulse voltammetry using MB as an electrochemical hybridization indicator. The fabricated electrochemical DNA biosensor was further used to analyze microRNA-21 in (spiked) human serum sample to investigate if it can be used as an electrochemical DNA biosensor for a wider range of applications that cover the fields of medical diagnostic and environmental monitoring.

## 2. Materials and Methods

### 2.1. Chemicals and Reagents

Carbon black (CB) N220 (32 nm) was obtained from Cabot Corporation (Ravenna, Italy). Dimethylformamide (DMF) was purchased from VMW International (Roquemaure, France). Chloroauric acid (HAuCl_4_) was purchased from Sigma-Aldrich (Saint Louis, MO, USA). Sulfuric acid (H_2_SO_4_) and potassium nitrate (KNO_3_) were obtained from Loba Chemie (Mumbai, India). 1-Ethyl-3-(3-dimethylaminopropyl)carbodiimide (EDC) was purchased from Merck (Darmstadt, Germany). Methylene blue (MB) was obtained from Breckland Scientific Supplies (Norfolk, UK). Bovine serum albumin (BSA) was obtained from VWR Life Science (Dublin, Ireland). Cysteamine (CysAm) and imidazole were obtained from Sigma-Aldrich (Buchs, Switzerland). All other chemicals and reagents were of analytical grade and used without further purification. All buffers were prepared in ultrapure water obtained from a Millipore Milli-Q purification system (18.2 MΩ·cm).

The synthesized and HPLC-purified oligonucleotides used in this study were obtained from Eurofins Genomics (Ebersberg, Germany). The oligonucleotide sequences are as follows: 5′–TCA ACA TCA GTC TGA TAA GCT A–3′ (complementary sequence of microRNA-21); 5′–UAG CUU AUC AGA CUG AUG UUG A–3′ (target microRNA-21 sequence); and 5′–UCC CUG AGA CCC UUU AAC CUG UGA–3′ (non-complementary microRNA-125a sequence). Stock solutions (100 µM) of all oligonucleotides were prepared with ultrapure water and kept frozen at −20 °C until use.

### 2.2. Apparatus and Instruments

The electrochemical measurements were carried out using PalmSens 2 Potentiostat obtained from PalmSens BV and the control and data acquisition were done through PSTrace 5.9 software from PalmSens BV (Houten, The Netherlands). The OriginPro 2022 (9.9) software from OriginLab (Northampton, MA, USA) was used for data analysis and graphing. The measurements were based on a conventional three-electrode system. The Ag/AgCl electrode was used as a reference electrode and a platinum wire was used as a counter electrode. The graphite lead (Faber-Castell HB 0.5 mm) was used as a working electrode called a pencil graphite electrode (PGE). The mechanical pencil (Pentel P250) was used as a holder for the graphite lead. The electrical contact with the lead was established by soldering a metallic wire to the metallic part of the mechanical pencil. The pencil lead was retained vertically with 1.5 cm of the lead protruding outside and 1 cm submerged in the solution. All measurements were performed at room temperature (20–25 °C).

The characterization with Fourier transform infrared (FTIR) spectroscopy was recorded with an IRAffinity-1S spectrophotometer (SHIMADZU) in the range of 2000–500 cm^−1^ at the attenuated total reflectance mode.

### 2.3. Preparation of AuNPs/CB Modified PGE

The PGE was pretreated in an acetate buffer solution (20 mM, pH 4.8) containing 20 mM NaCl and at an applied potential of +1.4 V during 60 s.

The pretreated PGE was incubated in 100 µL of CB dispersion during 1 h and kept drying for 20 min to obtain CB-modified PGE. The CB dispersion was prepared by sonicating 10 mg of CB in 10 mL of DMF for 1 h. The AuNPs synthesis was carried out onto the CB-modified PGE surface from an aqueous solution of 0.1 M KNO_3_ containing 2 mM of HAuCl_4_ by using cyclic voltammetry in a potential range from +0.9 to −0.3 V and at a scan rate of 50 mV·s^−1^ as reported in [53]. The AuNPs/CB-modified PGE was activated by running cyclic voltammetry in a potential range from +0.2 to +1.6 V in 0.5 M H_2_SO_4_ until a reproducible voltammogram was obtained and then the AuNPs/CB-modified electrode was washed with ultrapure water.

### 2.4. Preparation of Self-Assembled CysAm Monolayer and ssDNA Immobilization

**Scheme 1:**
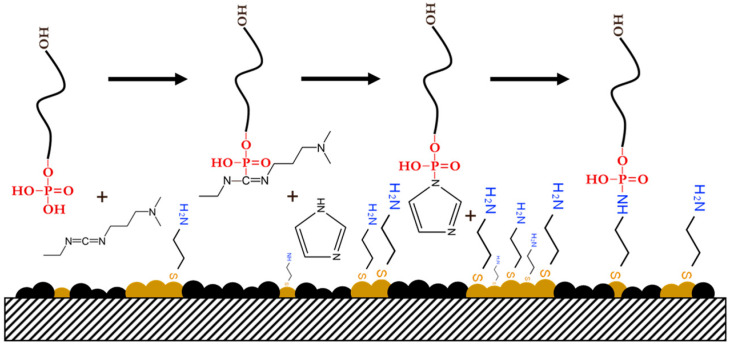
Attachment of the non-modified ssDNA capture probe from its 5′-terminal phosphate group to the CysAm-modified AuNPs/CB/PGE using a phosphoramidate-bonding strategy.

The AuNPs/CB-modified PGE was incubated in 100 µL of 0.1 M of CysAm and allowed to react for 2 h at room temperature. After that, the self-assembled CysAm monolayer with primary amine groups was formed and then the surface of the electrode was washed with ultrapure water before the ssDNA immobilization. The CysAm/AuNPs/CB-modified PGE was immersed in an imidazole buffer solution (0.1 M, pH 6.0) containing 0.1 M EDC and then allowed to react with the non-modified ssDNA overnight at 4 °C. The incorporation of imidazole with EDC allows the activation of the 5′-terminal phosphate group of the ssDNA capture probe by forming a highly reactive diester intermediate known as phosphorylimidazolide, as shown in Scheme 1. The reactive phosphorylimidazolide will react with the primary amine group of CysAm to form a phosphoramidate bond with the non-modified ssDNA capture probe.

The obtained ssDNA/CysAm/AuNPs/CB-modified PGE was washed several times with phosphate-buffered saline (PBS) (0.1 M, pH 7.4) to remove the unimmobilized ssDNA capture probe and then immersed in BSA solution 1% (*m*/*v*) for 20 min at room temperature to avoid nonspecific adsorption on the modified electrode surface.

### 2.5. MicroRNA-21 Hybridization and Electrochemical Measurements

The hybridization is carried out through the incubation of the obtained modified electrode referred to as BSA/ssDNA/CysAm/AuNPs/CB/PGE in various concentrations of microRNA-21 ranging from 1.0 × 10^−8^ to 5.0 × 10^−7^ M in PBS buffer (0.1 M, pH 7.4) and kept for 30 min at room temperature. The obtained electrode after hybridization referred to as miRNA-21/BSA/ssDNA/CysAm/AuNPs/CB/PGE was washed several times with the same buffer to remove the non-hybridized target sequences and then immersed into Tris-HCl buffer (20 mM, pH 7.00) containing 20 µM MB and 20 Mm NaCl to avoid the electrostatic interactions between MB and ssDNA or ssDNA-microRNA-21 hybrids. The accumulation of the electrochemical indicator was done for 5 min and then the electrode was washed with the same Tris-HCl buffer containing 20 mM of NaCl.

The reduction signal of accumulated MB was performed by using differential pulse voltammetry in a potential range from +0.1 to −0.8 V with an amplitude of 10 mV and at a scan rate of 50 mV·s^−1^ in Tris-HCl buffer (20 mM, pH 7.00) containing 20 mM of NaCl. The cyclic voltammetry measurements were conducted at a potential range from −0.2 to +0.65 V and at a scan rate of 50 mV·s^−1^ in 5 mM [Fe (CN)_6_]^3−/4−^ including 0.1 M KCl.

## 3. Results and Discussion

### 3.1. Electrochemical Characterization of Different Modified Electrodes

**Figure 1:**
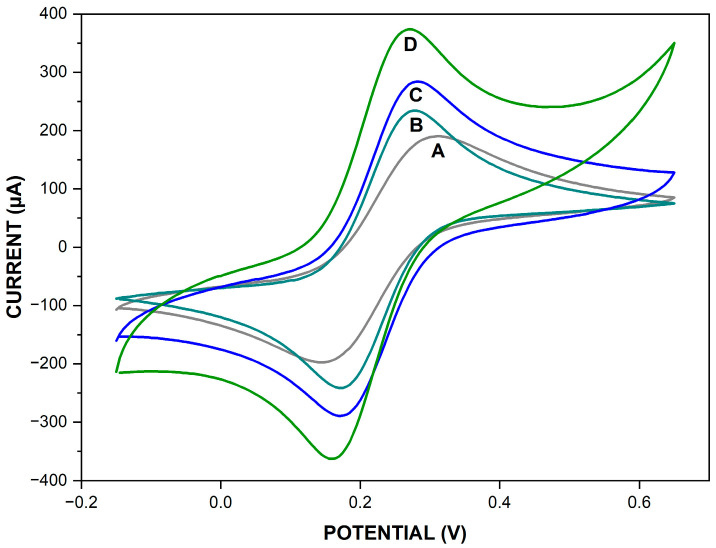
Cyclic voltammograms of bare PGE (A), CB/PGE (B), AuNPs/CB/PGE (C), and CysAm/AuNPs/CB/PGE (D) recorded in 5 mM [Fe(CN)_6_]^3−/4−^ containing 0.1 M KCl.

The cyclic voltammetry (CV) of different modified electrodes in 5 mM [Fe(CN)_6_]^3−/4−^ redox couple containing 0.1 M KCl at a scan rate of 50 mV·s^−1^ is shown in Figure 1. The bare PGE (A) shows a couple of quasi-reversible and well-defined redox peaks of [Fe(CN)_6_]^3−/4−^. After modification of the PGE with CB (B) and CB/AuNPs (C), the redox peak current of [Fe(CN)_6_]^3−/4−^ showed an increase, while the peak-to-peak separation was decreased compared to the bare PGE. These improvements could be attributed to the large specific surface area and good electron transfer capability of CB and AuNPs [23,26]. The redox peak current of [Fe(CN)_6_]^3−/4−^ increased even more after the subsequent modification of AuNPs/CB/PGE with CysAm (D). This increase could be explained by the electrostatic attraction between the positive-charged amine groups of CysAm and the negative-charged [Fe(CN)_6_]^3−/4−^ redox couple.

**Figure 2:**
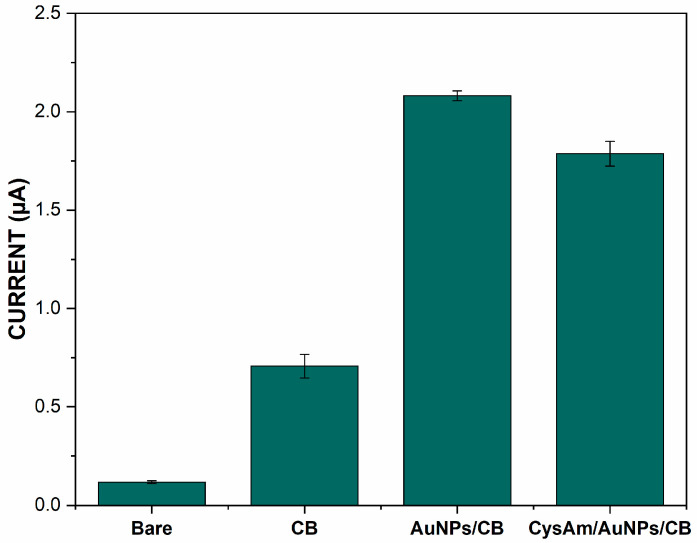
Differential pulse voltammetry reduction current of MB on bare PGE (RSD% = 5.55%), CB/PGE (RSD% = 8.43%), AuNPs/CB/PGE (RSD% = 1.19%), and CysAm/AuNPs/CB/PGE (RSD% = 3.73%) after incubation 20 µM MB at Tris-HCl buffer (20 mM, pH 7.00) including 20 mM NaCl for 5 min and washing with the same buffer including 20 mM NaCl (n = 3).

The properties of the modified electrodes were further investigated using the differential pulse voltammetry (DPV) reduction current of MB. Figure 2 shows the reduction current of MB for different modified electrodes after incubation in 20 µM of MB for 5 min at Tris-HCl (20 mM, pH 7.00) containing 20 mM of NaCl and washing with the same Tris-HCl buffer containing 20 mM of NaCl.

The bare PGE shows a low reduction current of MB. The reduction current of MB showed an increase compared to the bare PGE after the modification with CB and CB/AuNPs. These improvements could be attributed to the large specific surface area and good electron transfer capability of CB and AuNPs. The reduction current decreased after the subsequent modification of AuNPs/CB/PGE with CysAm. This decrease is due to the electrostatic repulsion between the positive-charged amine groups of CysAm and the MB. These results confirm those obtained with CV.

### 3.2. Characterization of ssDNA Immobilization and Microrna-21 Hybridization

**Figure 3:**
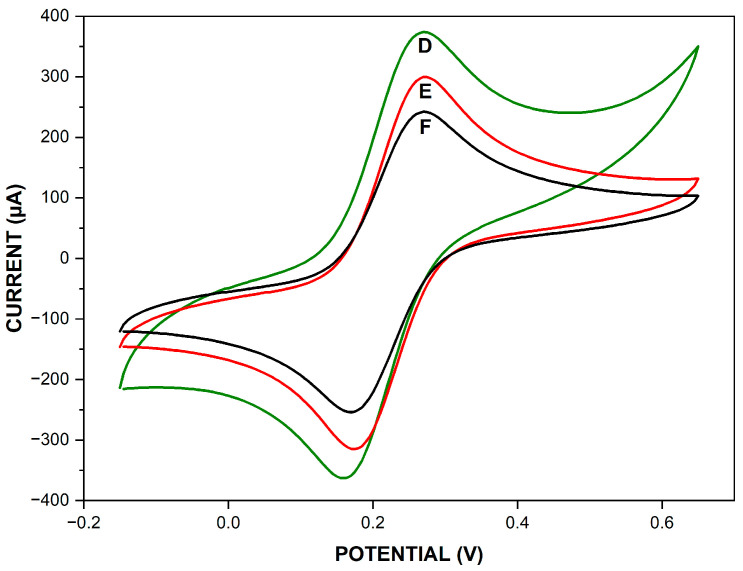
Cyclic voltammograms of CysAm/AuNPs/CB/PGE (D), BSA/ssDNA/CysAm/AuNPs/CB/PGE (E), and miRNA-21/BSA/ssDNA/CysAm/AuNPs/CB/PGE (F) recorded in 5 mM [Fe(CN)_6_]^3−/4−^ containing 0.1 M KCl.

The covalent immobilization of the ssDNA capture probe on the surface of CysAm/AuNPs/CB/PGE was realized through a phosphoramidate bond between the 5′-terminal phosphate group of ssDNA and the amine group of the electrode. The CV of CysAm/AuNPs/CB/PGE before (D) and after (E) covalent immobilization of non-modified ssDNA in 5.0 mM [Fe(CN)_6_]^3−/4−^ redox couple containing 0.1 M KCl at a scan rate of 50 mV·s^−1^ is shown in Figure 3. The high redox peak current at CysAm/AuNPs/CB/PGE is attributed, as mentioned before, to the electrostatic attraction between the positive-charged amine groups of CysAm and the negative-charged [Fe(CN)_6_]^3−/4−^ redox couple. The immobilization of the non-modified ssDNA capture probe on CysAm/AuNPs/CB/PGE caused a decrease in the redox peak current of [Fe(CN)_6_]^3−/4−^. This decrease is attributed to the electrostatic repulsion between the negative-charged phosphate backbone of the immobilized ssDNA capture probe and the negative-charged [Fe(CN)_6_]^3−/4−^ redox couple. This result suggests that the ssDNA capture probe has been effectively immobilized to the electrode surface. The redox peak current of [Fe(CN)_6_]^3−/4−^ decreased even further after the hybridization between the immobilized ssDNA capture probe and their complementary target sequence (F). This decrease is related to an increase in the negative-charged phosphate backbone resulting from the formation of ssDNA-microRNA-21 hybrids.

**Figure 4:**
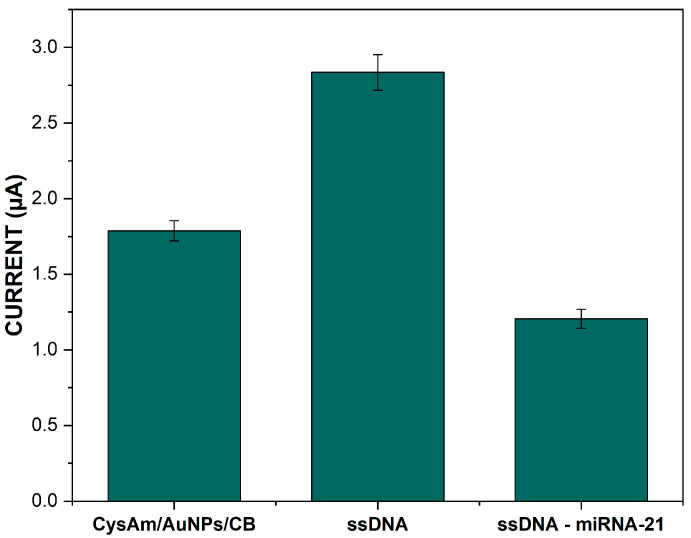
Differential pulse voltammetry reduction current of MB on CysAm/AuNPs/CB/PGE (RSD% = 3.73%), BSA/ssDNA/CysAm/AuNPs/CB/PGE (RSD% = 4.18%), and miRNA-21/BSA/ssDNA/CysAm/AuNPs/CB/PGE (RSD% = 5.24%) after incubation in 20 µM of MB at Tris-HCl buffer (20 mM, pH 7.00) including 20 mM NaCl for 5 min and being washed with the same buffer including 20 mM NaCl (n = 3).

The covalent immobilization of the ssDNA capture probe was further investigated using the differential pulse voltammetry reduction current of MB. Figure 4 shows the reduction current of MB before and after covalent immobilization of the ssDNA capture probe. The primary reduction current at CysAm/AuNPs/CB/PGE is attributed, as mentioned before, to the ability of CB and AuNPs to promote more electron transfers and to the large specific surface area. The immobilization of the ssDNA capture probe on CysAm/AuNPs/CB/PGE caused an increase in the reduction current of MB. This increase in the reduction current is actually due to the strong affinity between MB and the free guanine bases of the ssDNA capture probe. This result also confirms that the ssDNA capture probe has been effectively immobilized on the electrode surface.

**Figure 5:**
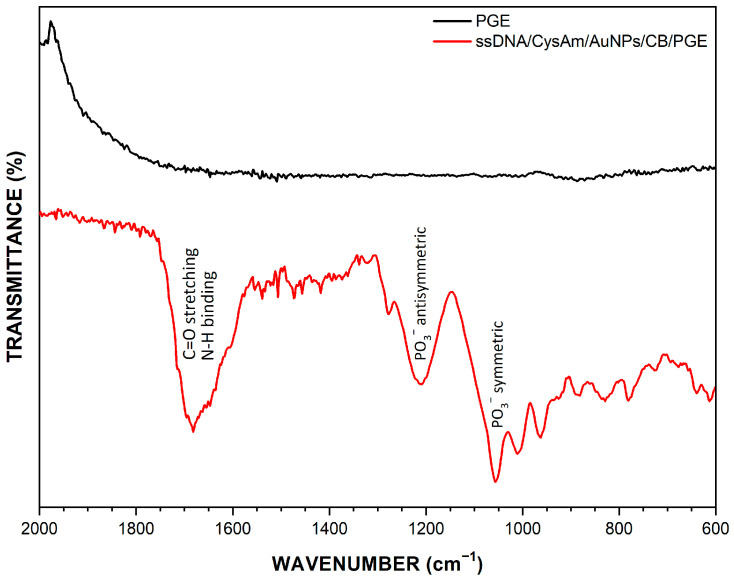
FTIR spectra of immobilized ssDNA capture probe on the CysAm/AuNPs/CB/PGE.

The reduction current of MB was decreased after the hybridization between the immobilized ssDNA capture probe and its complementary microRNA-21 target sequence, as shown in Figure 4. This decrease is due to the ssDNA-microRNA-21 hybrids preventing the interaction between MB and guanine bases of the ssDNA capture probe. The differential pulse voltammetry results were in good agreement with those from CV. Both results of DPV and CV indicated that the electrochemical DNA biosensor has been successfully fabricated. Further investigation was done through infrared spectroscopy from 2000–500 cm^−1^ as shown in Figure 5.

The region between 1550 and 1750 cm^−1^ has stronger bands that correspond to the N-H bending and double-bond stretching vibrations of DNA bases. Peaks around 1100 and 1245 cm^−1^ are attributed to the symmetric and antisymmetric phosphate stretching vibrations of the phosphate backbone of the immobilized ssDNA capture probe. These results are in good agreement with those from the CV and DPV, as well as with the observed vibrational modes from surface-immobilized ssDNA reported in the literature [54].

### 3.3. Optimization of the Immobilized ssDNA Capture Probe Concentration

**Figure 6:**
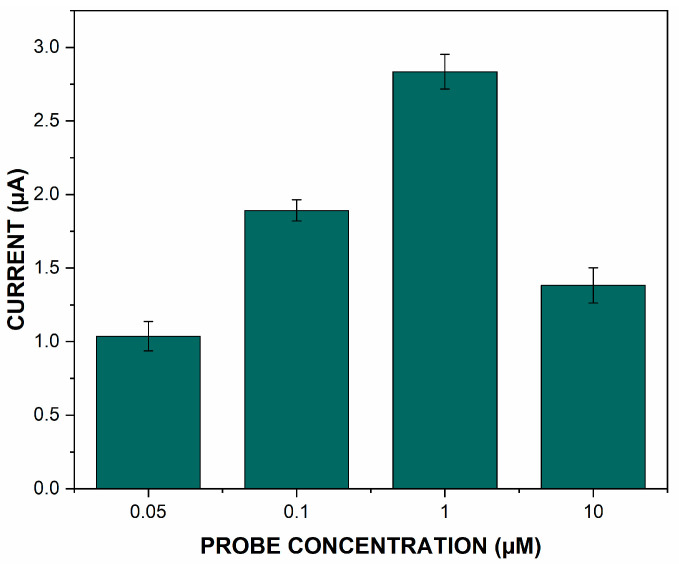
Differential pulse voltammetry reduction current of MB on CysAm/AuNPs/CB/PGE after immobilization of different concentrations of the ssDNA capture probe from 0.05 to 10 µM (RSD% = 9.69%, 3.81%, 4.18%, 8.68%). Incubation in 20 µM MB at Tris-HCl buffer (20 mM, pH 7.00) including 20 mM NaCl for 5 min and washing with the same buffer including 20 mM NaCl (n = 3).

The effects of ssDNA capture probe concentration on the performance of the constructed biosensor were investigated. Figure 6 shows the reduction current of MB after the covalent immobilization of different concentrations of the ssDNA capture probe. The reduction current of MB increased with the increase in ssDNA capture probe concentration from 0.1 to 1 µM. This increase was actually due to the strong affinity between MB and the free guanine bases related to the immobilized concentration of the ssDNA capture probe. The reduction current of MB decreased beyond the ssDNA capture probe concentration of 1 µM. This decreased reduction current of MB observed at the ssDNA capture probe concentration of 10 µM could be due to the steric hindrance caused by the larger quantity of the immobilized ssDNA capture probe. The reduction current of MB obtained with the ssDNA capture probe concentration of 0.05 µM is less than the previously reported at the surface of CysAm/AuNPs/CB/PGE. This obtained reduction current of MB is due to the large free surface without ssDNA capture probes. This large free surface is blocked with BSA, which consequently decreases the electron transfer. The ssDNA capture probe concentrations of 0.1 and 1 µM were consequently evaluated to obtain the most optimal hybridization response. Both ssDNA probe concentrations of 0.1 and 1 µM are evaluated with three different concentrations of microRNA-21 to determine the most appropriate probe concentration for the quantitative analysis.

**Figure 7:**
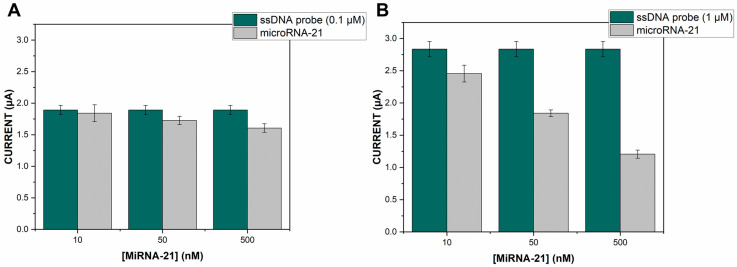
Differential pulse voltammetry reduction current of MB on BSA/ssDNA/CysAm/AuNPs/CB/PGE after the immobilization of 0.1 µM (**A**) (RSD% = 7.20%, 3.76%, 4.25%) and 1 µM (**B**) (RSD% = (5.28%, 2.75%, 5.24%) of ssDNA capture probe and the incubation with 10, 50, and 500 nM of microRNA-21 target concentration. Incubation in 20 µM MB at Tris-HCl buffer (20 mM, pH 7.00) including 20 mM NaCl for 5 min and washing with the same buffer including 20 mM NaCl (n = 3).

The capture probe concentration of 0.1 µM shows no significant difference before and after hybridization with 10, 50, and 500 nM of microRNA-21 target sequence concentrations (Figure 7A). This result could be attributed to the small amount of the immobilized ssDNA capture probe, which does not allow good hybridization even under the saturating concentration of microRNA-21. The ssDNA capture probe concentration of 1 µM shows a significant and proportional difference (Figure 7B). This is due to the sufficient concentration of the ssDNA capture probe, which allows a good and efficient hybridization with microRNA-21 target sequence concentrations. The ssDNA probe concentration of 1 µM was selected as the optimal capture probe concentration according to the MB reduction current obtained results.

### 3.4. Hybridization Selectivity and the Quantitative Analysis

**Figure 8:**
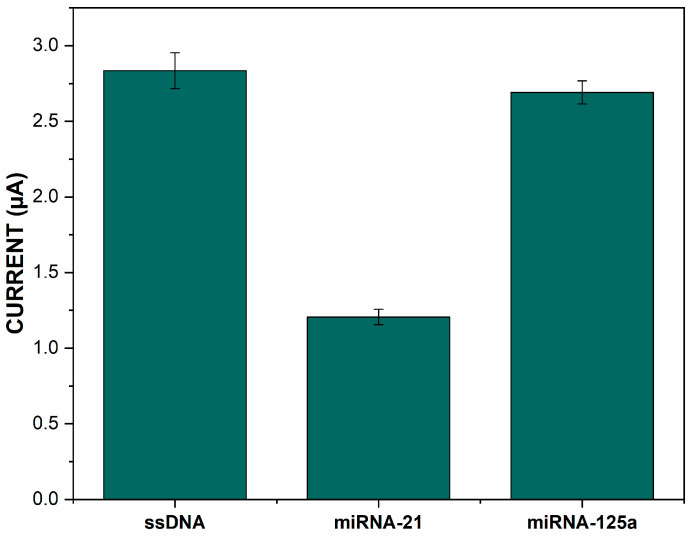
Differential pulse voltammetry reduction current of MB on BSA/ssDNACysAm/AuNPs/CB/PGE before and after hybridization with complementary (microRNA-21) (RSD% = 2.75%) and non-complementary (microRNA-125a) sequences (RSD% = 2.81%). Incubation in 20 µM MB at Tris-HCl buffer (20 mM, pH 7.00) including 20 mM NaCl for 5 min and washing with the same buffer including 20 mM NaCl (n = 3).

The selectivity of the fabricated biosensor was evaluated through hybridization between the capture probe and its complementary and non-complementary (microRNA-125a) sequence. The change in the reduction current of MB after hybridization with complementary and non-complementary target sequences is shown in Figure 8. The reduction current of MB was decreased using the complementary target sequence and it obviously shows that hybridization occurs successfully. The reduction current of MB was not changed significantly after the interaction with the non-complementary target sequence and it indicates that there is no successful hybridization. This means that the surface properties of the fabricated biosensor remained unchanged after its interaction with the non-complementary target sequence. These results demonstrate that the fabricated biosensor has good selectivity for discriminating differences between microRNA-21 and other non-complementary target sequences.

**Figure 9:**
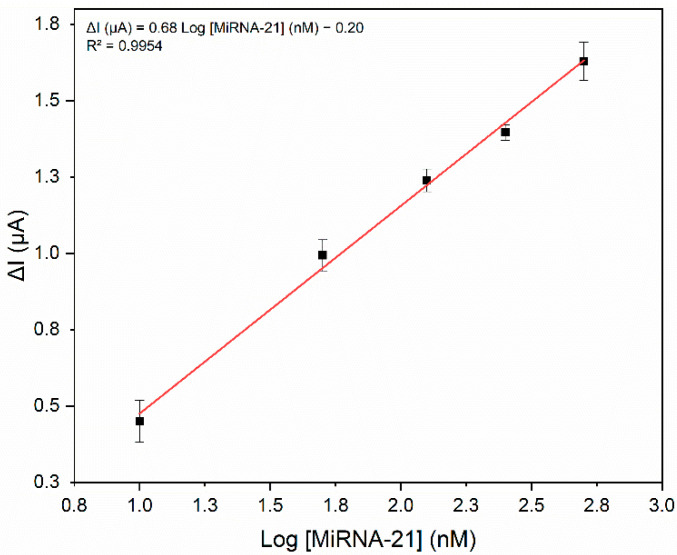
Calibration curve of ΔI value of current response of MB as a function of different microRNA-21 target concentration (from 1.0 × 10^−8^ to 5.0 × 10^−7^ M). The concentration of the capture probe was 1 µM. Measurements (DPV) were done in Tris–HCl buffer (20 mM, pH 7.0) including 20 mM of NaCl.

The quantitative analysis capability of the fabricated biosensor was also evaluated using different concentrations of microRNA-21. The change in the reduction current of MB (ΔI) after hybridization with different concentrations of microRNA-21 is shown in Figure 9. The ΔI was found to be well-proportional to the logarithmic value of the microRNA-21 concentration ranging from 1.0 × 10^−8^ to 5.0 × 10^−7^ M. The regression equation is ΔI = 0.68 × Log [miRNA-21] − 0.20 (R^2^ = 0.9954) and the detection limit was estimated to be 1 nM (3σ). These results showed that the fabricated biosensor was good enough for the sensitive detection of microRNA-21 and showed encouraging results as a platform to use for other specific sequences related to some well-known diseases.

The developed electrochemical DNA biosensor reproducibility was studied using three parallel sensors known as CysAm/AuNPs/CB/PGE and modified with 1 µM of ssDNA probe and BSA (1%) and at two different concentrations of microRNA-21. At a small concentration of microRNA-21 (1.0 × 10^−8^ M), the RSD% (n = 3) was equal to 2.91% and at high concentration (5.0 × 10^−7^ M) the RSD% was equal to 5.24%, and this show that the fabricated electrochemical DNA biosensor has a satisfactory reproducibility for microRNA-21 detection.

There are, as discussed before and as shown in Table 1, several ssDNA immobilization strategies developed in recent years and they are all based on three important mechanisms: physical adsorption, biotin-avidin affinity interaction, and covalent bonding. Physical adsorption is the simplest immobilization method because it does not require any prior modification of ssDNA. In this case, the immobilization is based on electrostatic interaction occurring between the negative-charged phosphate backbone of ssDNA and the positive charges covering the surface of the sensor. The limitation of the physical adsorption method is the random orientation and weak attachment of ssDNA to the surface, as noted before. The covalent immobilization gives us good vertical orientation and high binding strength, but it presents also some limitations such as the need to modify the ssDNA with some functional groups such as the thiol [-SH], amine [-NH_2_], and carboxylic [-COOH] groups. The immobilization strategy presented in this work combines the advantages of physical adsorption and the covalent bonding and leads to the immobilization of the ssDNA capture probe without need for any prior modification and with vertical orientation and good binding capacity.

The obtained limit of detection, in contrast, seems to be relatively low and is, in consequence, more suitable for specific disease biomarkers, as well as for the monitoring of particular environmental pollutants. This introduced immobilization strategy, as well as using pencil graphite lead as a sensor, could be adapted with modern amplification strategies based on enzymes [59] and nanozymes [60] for the detection of a wider range of disease biomarkers and for the monitoring of additional environmental pollutants.

### 3.5. Analysis of microRNA-21 in Serum Sample

The analysis of microRNA-21 was performed in diluted serum (20×). Different concentrations of MiRNA-21 were added to the human serum sample. Table 2 shows the average ΔI current obtained for three different concentrations of microRNA-21. The obtained results in Table 2 also showed a recovery of 98.4–102.13%.

These results suggest that the fabricated DNA biosensor has an acceptable detection performance in (spiked) human serum and it can be practically applied to detect microRNA-21 in real samples.

## 4. Conclusions

In conclusion, we have reported a simple and cost-effective alternative approach to the traditionally used covalent immobilization strategies. The presented method is based on the covalent immobilization of a non-modified ssDNA through a phosphoramidate bond between the 5′-terminal phosphate group of ssDNA and the amine-modified electrode surface so as to develop electrochemical DNA biosensors. The microRNA-21 was used as a model in this work, and the encouraging results obtained on human serum sample using a modified pencil graphite lead as a sensor open the way for further developments and applications such as the fabrication of cheap and innovative electrochemical DNA biosensor devices. This immobilization method, as well as the used pencil graphite lead, can be adapted with modern amplification strategies for the detection of a wider range of disease biomarkers and for the monitoring of environmental pollutants.

## Figures and Tables

**Table 1:** Comparison of different strategies of ssDNA probe immobilization on different matrix.

Immobilization Matrix	Immobilization Technique	Chemical Interaction	DNA Modification	Advantages	Drawbacks	LOD (M)	Ref.
Poly-L-Lysine	Adsorption	Electrostatic adsorption	No modification	Simple, fast, and direct (no linker molecules)	Desorption risk and random orientation	4.4 × 10^−9^	[49]
Chitosan						1.6 × 10^−11^	[55]
Avidin-Chitosan/MWCNTs	Avidin-Biotin	Avidin-Biotin affinity	Biotin-modified DNA	Improved orientation, high specificity, and reversible	Use of biocompatible linker and expensive	1.0 × 10^−16^	[56]
Avidin-MWCNTs						1.5 × 10^−7^	[57]
GTD/Chitosan-MWCNTs	Covalent	Aldimine bond	Amine-modified DNA	Good orientation, high binding strength, and adaptative linker	Use of linker molecules and expensive	8.5 × 10^−14^	[33]
Cysteine film		Au-S bond	Thiol-modified DNA			18 × 10^−8^	[58]
CysAm/AuNPs/CB	Covalent	Phosphoramidate bond	No modification	Good orientation, high binding strength, and no linker molecules needed	Reaction time and irreversible	1 × 10^−9^	This work

**Table 2:** Analytical results for microRNA-21 detection in (spiked) diluted (20×) human serum sample using the fabricated electrochemical DNA biosensor (n = 3).

Added (nM)	Detected (nM)	ΔI (µA)	Recovery (%)	RSD (%)
10	9.84	0.443	98.4	2.15
50	47.37	0.942	94.74	2.75
125	127.66	1.309	102.13	2.87
